# Damping Force and Loading Position Dependence of Mass Sensitivity of Magnetoelastic Biosensors in Viscous Liquid

**DOI:** 10.3390/s19010067

**Published:** 2018-12-25

**Authors:** Kewei Zhang, Zhe Chen, Qianke Zhu, Yong Jiang, Wenfeng Liu, Peixuan Wu

**Affiliations:** 1 School of Materials Science and Engineering, Taiyuan University of Science and Technology, Taiyuan 030024, China; drchenzhe@163.com (Z.C.); drzhuqianke@126.com (Q.Z.); yjiang@ustb.edu.cn (Y.J.); WFLiu@tyust.edu.cn (W.L.); 2 The Key Laboratory of Magnetic and Electric Functional Materials and Applications of Shanxi Province; 3 Guangdong Provincial Key Laboratory of Micro-nano Manufacturing Technology and Equipment, Guangdong University of Technology, Guangzhou, Guangdong 510006, China; peixuan@gdut.edu.cn

**Keywords:** magnetoelastic, biosensor, sensitivity, damping force

## Abstract

We established the vibration governing equation for a magnetoelastic (ME) biosensor with target loading in liquid. Based on the equation, a numerical simulation approach was used to determine the effect of the target loading position and viscous damping coefficient on the node (“blind points”) and mass sensitivity (*S*_m_) of an ME biosensor under different order resonances. The results indicate that viscous damping force causes the specific nodes shift but does not affect the overall variation trend of *S*_m_ as the change of target loading position and the effect on Sm gradually reduces when the target approaches to the node. In addition, *S*_m_ decreases with the increase of viscous damping coefficient but the tendency becomes weak at high-order resonance. Moreover, the effect of target loading position on *S*_m_ decreases with the increase of viscous damping coefficient. Finally, the results provide certain guidance on improving the mass sensitivity of an ME biosensor in liquid by controlling the target loading position.

## 1. Introduction

In recent years, there has been increasing interest in magnetoelastic (ME) biosensors due to the advantages of low cost, easy operation, wireless, and real-time detection [1]. An ME biosensor is comprised of a free-standing strip-shaped sensor platform made of ferromagnetic materials (Metglas 2826MB alloy) with a layer of bio-probes (antibody, phages) immobilized on its surface. The working principle is based on the change in resonant frequency of an ME biosensor in response to the specific binding between the target and the bio-probes (mass load of the target) under an alternative magnetic field [2]. To date, ME biosensors have been successfully developed for the detection of food-borne pathogens, virus, chemicals and heavy metal ions such as *Escherichia coli* [3], *Listeria monocytogens* [3], *Salmonella Typhimurium* [3,4], *Bacillus anthracis spores* [4,5], *Staphylococcus aureus* [3,6], *Staphylococcus epidermidis* [7], *swine fever virus* [8], *uranyl* [9], Pb^2+^, Cd^2+^, Cu^2+^ and Hg^2+^ [10,11] in water. Particularly, the development of portable resonant signal interrogation devices [12,13,14,15] and direct detection of pathogens on the surface of spinach leaves [16], tomatoes [17] and eggshells [18] makes in-situ detection of ME sensors to be possible. 

For an ME biosensor, a key parameter to characterize its performance is mass sensitivity (*S*_m_) which is defined as the change of resonant frequency caused by per unit load mass (the target mass) [19]. In other words, high *S*_m_ means large shift of resonant frequency for the same target loading. However, the *S*_m_ for an ME biosensor still needs to be improved to meet the future requirement for the detection of a small number of targets or even single target. Although the *S*_m_ of an ME biosensor can be improved by reducing its size [1], the signal intensity decreases along with the sensor size. Another way to improve the *S*_m_ is to choose the sensor platform materials with high Young’s modulus and low density [1] but other properties such as magneto-mechanical coupling coefficient and magnetic permeability also need to be considered. It has been experimentally found that target distribution on an ME biosensor plays a decisive role on the *S*_m_. Especially, the *S*_m_ is zero when the target is loaded at the node (also called “blind point”) of an ME biosensor [20]. In addition, it has been found that the “blind point” issue can be overcome by using multiple mode resonant frequencies [20]. In our previous studies, we established the vibration governing equation for an ME biosensor loaded with a target in different positions and revealed the variation of *S*_m_ as the function of target position or distribution by mathematical simulation [21,22,23,24]. The results suggest that the *S*_m_ is linearly proportional to the square of point displacement where the target is loaded. Unfortunately, all the aforementioned studies are suitable only for the detection in air, whereas much detection in reality has to be carried out in liquid. In this case, extra viscous damping force is introduced to an ME biosensor that may affect its overall resonant behavior as well as the mass sensitivity. Chen et al. [25] studied the resonance behavior of a rod-like cantilever vibrating in a viscous liquid and determined a closed-form solution for the added mass and damping coefficient. In the work, they treated the viscous damping force as virtual mass. However, the variation of mass sensitivity as the mass loading position is not given. 

The aim of this work was to address the above issue by establishing the vibration governing equation of a free-standing ME biosensor with target loading in liquid and find the variation rule of mass sensitivity as the change of target loading position and viscous damping force under different resonance modes.

## 2. Determination of Materials, Liquid and Loading Conditions

**Figure 1:**
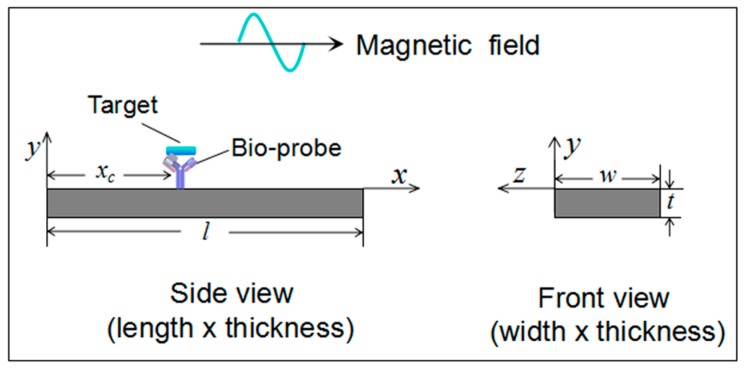
Schematic illustration of a target loaded magnetoelastic sensor vibrating in a liquid. *l*, *w*, and *t* are the total length, width and thickness of the sensor, respectively; and *x_c_* is *x*-coordinate of the target.

Assume the target is loaded on an ME biosensor that is vibrating in a liquid, as shown in Figure 1. Here, we chose the commercial available ferromagnetic alloy Metglas 2826MB (Fe_40_Ni_38_Mo_4_B_18_) as the sensor platform materials, which has been widely used in ME biosensors. The detailed information of the sensor, liquid and the target loading conditions for this study is listed in Table 1.

## 3. Establishment of Vibration Governing Equation

Based on the above assumption, the kinetic energy (*T*), potential energy (*V*) of the sensor and the dissipative function (*D*) can be expressed as:

(1) $$T=\frac{1}{2}\int_{0}^{l}\rho A{\left(\frac{\partial u\left(x,t\right)}{\partial t}\right)}^{2}dx+\frac{1}{2}m{\left(\frac{\partial u\left(x,t\right)}{\partial t}\right)}_{x=x_{c}}^{2}$$

(2) $$V=\frac{1}{2}\int_{0}^{l}\frac{E}{1-\nu }A{\left(\frac{\partial u\left(x,t\right)}{\partial x}\right)}^{2}dx$$

(3) $$D=\frac{1}{2}\int_{0}^{l}c_{l}{\left(\frac{\partial u\left(x,t\right)}{\partial t}\right)}^{2}dx$$

where *ρ*, *E*, *ν*, and *A* are density, Young’s modulus, Poisson’s ratio and cross-sectional area (*w* × *t*) of the sensor, respectively; *m* represents mass of the target; *c_l_* represents the viscous damping coefficient per unit length; and *u* is the point displacement at *x*, which is expressed as:

(4) u(x,t)=ϕ(x)γ(t)

with *φ*(*x*) being the mode shape function, which is assumed as $\varphi \left(x\right)=cos\frac{n\pi x}{L}$ (*n* = 1,2,3), where *n* is the mode number and *γ* is the generalized coordinate. For the *n*th-order mode in Equation (4), the point displacement function is $u\left(x,t\right)=u_{n}\left(x,t\right)=\varphi_{n}\left(x\right)\gamma_{n}\left(t\right)$.

By substituting Equation (4) into Equations (1)–(3), we can obtain that:

(5) $$T=\frac{1}{2}{\dot{\gamma }}^{T}\left(M_{s}+M_{c}\right)\dot{\gamma }=\frac{1}{2}{\dot{\gamma }}^{T}M\dot{\gamma }$$

(6) $$V=\frac{1}{2}\gamma^{T}K\gamma$$

(7) $$D=\frac{1}{2}{\dot{\gamma }}^{T}c\dot{\gamma }$$

where $M_{s}=\int_{0}^{l}\rho A\phi^{T}\phi dx$, $M_{c}=m\phi^{T}\phi$, $M=M_{s}+M_{c}$, $K=\int_{0}^{l}\frac{E}{1-\nu }A\phi^{\prime T}\phi^{\prime }dx$, $C=\int_{0}^{l}c_{l}\phi^{T}\phi dx$.

It is known that Lagrange’s equation for a system with dissipative force can be expressed as:

(8) $$\frac{d}{dt}\left(\frac{\partial L}{\partial \dot{\gamma }}\right)-\left(\frac{\partial L}{\partial \gamma }\right)+\frac{\partial D}{\partial \dot{\gamma }}=F$$

where *L* = *T* − *V* and *F* is a generalized force that is in the form of $F=\int_{0}^{l}\varphi^{T}fdx$.

After inputting Equations (5)–(7) into Equation (8), we can get the vibration governing equation as:

(9) $$M\ddot{\gamma }+c\dot{\gamma }+K\gamma =F$$

Unfortunately, Equation (9) is undecoupled and thus it is difficult to obtain the particular solution *γ*. To decouple Equation (9), we normalize *φ_n_* to $\psi_{n}=\left(\frac{\phi_{n}}{\sqrt{\phi_{n}^{T}M\phi_{1}}}\right)$ and let γ=ψq. Then, Equation (9) can be converted as:

(10) $$M\psi \ddot{q}+C\psi \dot{q}+K\psi q=F$$

By multiplying $\psi^{T}$ on the both sides of Equation (10), we can obtain a decoupled vibration governing equation as expressed as:

(11) $${\ddot{q}}_{n}+C_{n}{\dot{q}}_{n}+K_{n}q_{n}=Q_{n}$$

where $I_{n}=\psi_{n}^{T}M\psi_{n}\ddot{q}$, $C_{n}=\psi_{n}^{T}C\psi_{n}$, $K_{n}=\psi_{n}^{T}K\psi_{n}$, $Q_{n}=\psi_{n}^{T}F$.

Here, *I_n_*, *C_n_*, *K_n_*, and *Q_n_* are all diagonal matrices.

According to the fundamentals of mechanical vibrations, the particular solution *q_n_* of Equation (11) is known as:

(12) $$q_{n}=\frac{Q_{n}}{\omega_{n}^{2}\sqrt{{\left(1-\lambda_{dn}^{2}\right)}^{2}+{\left(2\xi_{n}\lambda_{dn}^{2}\right)}^{2}}}\sin(\omega t-\phi_{n})$$

where $\omega_{n}=\sqrt{\frac{K_{n}}{M_{n}}}$, $\xi_{n}=\frac{C_{n}}{2\omega_{n}}$, $\lambda_{dn}=\sqrt{1-2\xi_{n}^{2}}$.

After substituting Equation (12) into Equation (11), we can determine the *ω*_dn_ for a given condition, and the mass sensitivity can be obtained by

(13) $$S_{m}=\frac{\Delta f_{dn}}{m}=\frac{f_{dnm}-f_{dn}}{m}=\frac{\omega_{dnm}-\omega_{dn}}{2\pi m}$$

where *f*_dn_ and *f*_dnm_ represent the displacement resonance frequency of the sensor without and with target loading; and $\omega_{dn}=\omega_{n}\sqrt{1-\xi_{n}^{2}}$, which represents the displacement resonant angular frequency.

## 4. Results and Discussion

### 4.1. Effect on Vibration Mode Shape and Nodes

**Figure 2:**
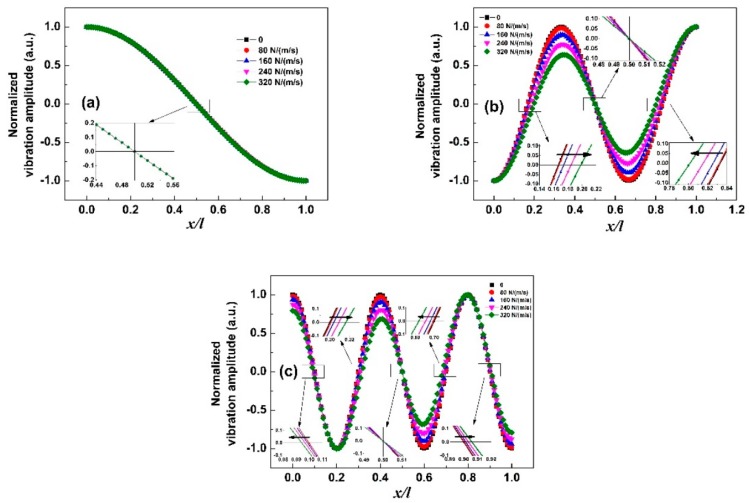
Vibration mode shapes of the sensor in air and in liquid with different viscous damping coefficient: (**a**) first-order resonance; (**b**) third-order resonance; and (**c**) fifth-order resonance. Here, the target loading position *x*_c_/*l* = 0.1 is used as an example.

**Figure 3:**
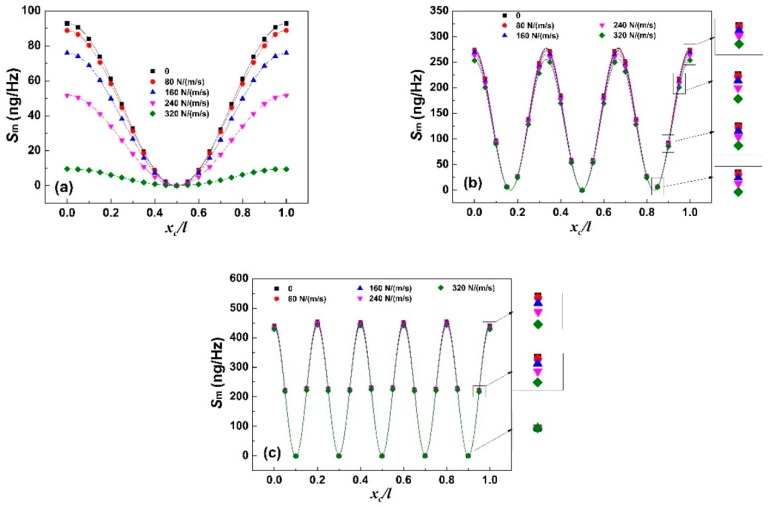
*S*_m_ as the function of target loading position in the liquids with different viscous damping coefficient under: (**a**) first-order resonance; (**b**) third-order resonance; and (**c**) fifth-order resonance.

From Equation (11), we can see that the extra damping force in liquid affects the vibration governing equation and thus causes the change of vibration mode shape as well as the mass sensitivity *S*_m_. Figure 2 shows the vibration mode shapes of the sensor in different liquid with the target loaded at *x*_c_/*l* = 0.1. In Figure 2a, we can see that the curves almost overlap suggesting that the viscous damping force has little effect on the vibration mode shape for the first-order resonance. However, the curves in Figure 2b,c exhibit significant difference, indicating the increasing effect at higher-order resonance. In addition, it was found that there are n nodes for the nth-order resonance and the node(s) for each mode of resonance shift with the change of viscous damping coefficient except for the one at the middle of the sensor. In detail, only one node at the middle of the sensor does not shift with the change of *c* for the first-order resonance. For the third-order resonance, the three nodes from left to right shift toward positive, zero, and negative *x*-axis direction, respectively, with the increase of viscous damping coefficient, as shown as the inset in Figure 3b. For the fifth-order resonance, the five nodes shift toward negative, positive, zero, negative, and positive direction, respectively, as shown as the inset in Figure 3c. Since the neighbor of each node is a mass insensitive region, the shift of a node means that the insensitive region moves consequently.

### 4.2. Effect on Mass Sensitivity

Figure 3 shows the mass sensitivity *S*_m_ of the sensor as the function of target loading position in air and in liquid. Clearly, the extra dissipative force decreases the overall mass sensitivity for all resonance modes but this effect becomes weaker with the increase of resonance order. Besides, all curves exhibit a sine-wave shape and *n* + 1 local maximal values with *n* local minimal values are found in each curve, where *n* is the corresponding resonance order. Combining with the results in Figure 2, the local minimal *S*_m_ at *x*_c_/*l* = 0.5 for all curves in Figure 3 is zero, which means it is unrelated to the resonance order and viscous damping coefficient. However, the other local minimal values of *S*_m_ with the corresponding *x*_c_/*l* are different with the change of viscous damping coefficient or resonance order. Furthermore, it is worth noting that viscous damping coefficient does not affect the variation trend of *S*_m_ with the change of loading position but weakens the effect of loading position on *S*_m_ and the weakening effect increases with the increase of viscous damping coefficient.

**Figure 4:**
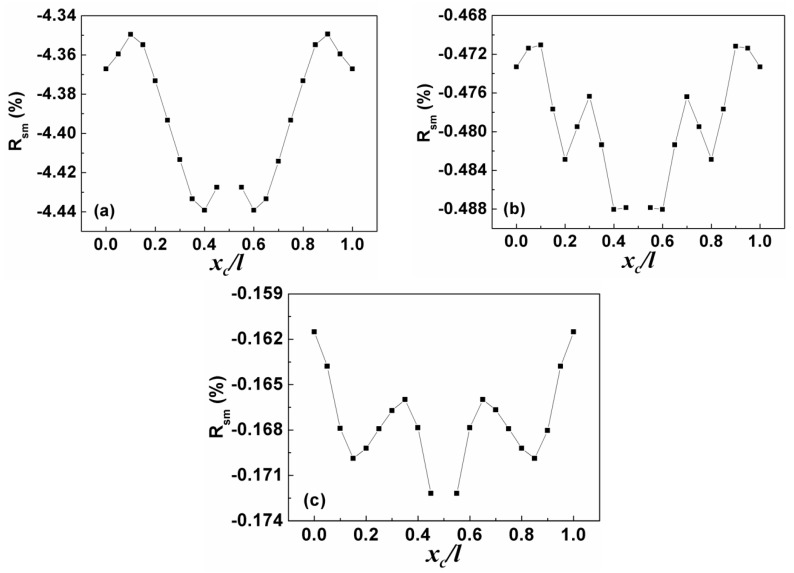
*R_sm_* as the function of loading position in the liquid with viscous damping coefficient of 80 N/(m/s) under: (**a**) first-order resonance; (**b**) third-order resonance; and (**c**) fifth-order resonance.

**Figure 5:**
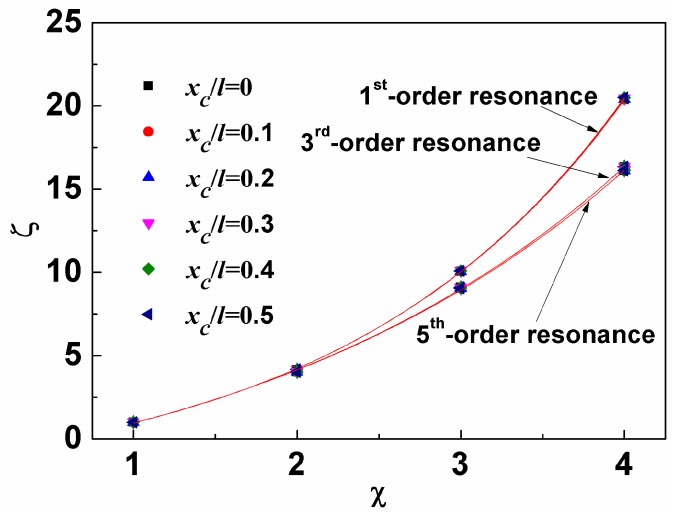
*R_sm_* ratio (*ζ*) as the function of viscous damping coefficient ratio (*χ*).

Figure 4 shows *R*_sm_ as a function of loading position in Liquid #1 where *R*_sm_ = (*S*_m,liquid_ − *S*_m,air_)/*S*_m,air_. It was observed that all the curves exhibit a complex “M+W” shape, in which the singular point at *x*_c_/*l* = 0.5 is due to S_m,air_ = 0, as aforementioned. In addition, the absolute value of *R*_sm_ for the same loading position decreases with increasing resonance order, which again indicates the less viscous damping coefficient dependence of mass sensitivity for the sensor at higher order resonance. The curves for the other liquids show a similar behavior but with a decreasing value of *R*_sm_ as the viscous damping coefficient increases, as shown in Figure 5, where the *x*-axis and *y*-axis represent the viscous damping coefficient ratio (*ζ*) and *R*_sm_ ratio (*χ*) of Liquids #2, #3, and #4 to Liquid #1, respectively. We found that the data in the figure for the same order resonance can be well fitted by the equation and the nearly overlapping curves indicate that the loading position has much less effect on *R*_sm_ compared to viscous damping coefficient. In addition, the significant difference between the curves for the first-order resonance and higher-order resonance again suggests the weak effect of viscous damping coefficient on mass sensitivity at high-order resonance.

**Figure 6:**
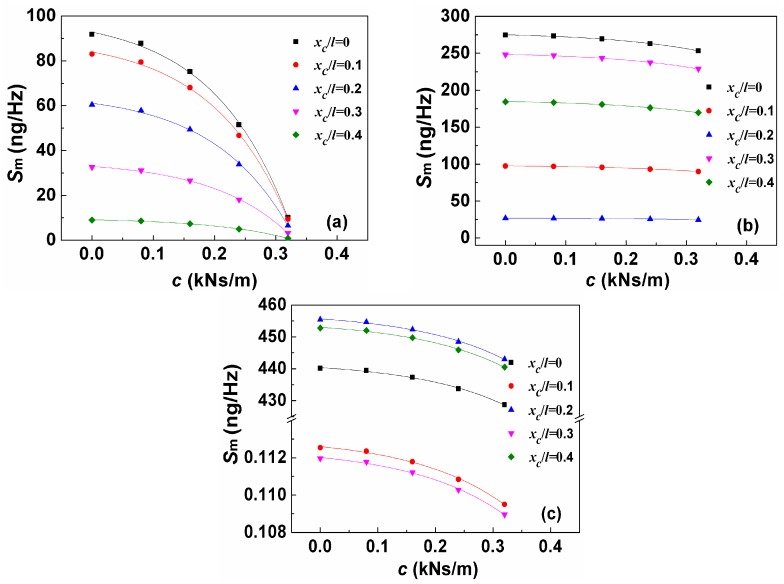
*S*_m_ as the function of viscous damping coefficient *c* for different loading positions under: (**a**) first-order resonance; (**b**) third-order resonance; and (**c**) fifth-order resonance.

Figure 6 shows the *S*_m_ as a function of viscous damping coefficient for different loading positions. We can see that all data for the same loading position can be well fitted by an exponent decaying function. In other words, with the increase of viscous damping coefficient, *S*_m_ decreases and tends to approach each other but the tendency becomes weaker at high-order resonance. In addition, when the target is loaded close to the nodes, the contribution of viscous damping coefficient to the decrease of *S*_m_ gradually reduces. Taking the fifth-order resonance as an example, the slopes of the curves for *x*_c_/*l* = 0.1, 0.3, and 0.5 are close to or equal to zero, which is due to the three nodes near *x*/*l* = 0.1, 0.3, and 0.5, respectively.

## 5. Conclusions

We studied the resonance behavior and mass sensitivity (*S*_m_) of a magnetoelastic (ME) biosensor with target loading in liquids with viscous damping coefficient in the range of 80–320 N/(m/s). Several conclusions were drawn as follows:

1. Viscous damping force causes the vibration mode shapes to change, which results in the nodes shifting except for the one at the middle of the sensor and the change becomes more obvious at higher-order resonance.
2. Viscous damping force does not affect the variation trend of *S*_m_ as the change of target loading position but will weaken the effect of loading position on *S*_m_ and the weakening effect increases with the increase of viscous damping coefficient. *n* + 1 local maximal sensitivity and *n* local minimal sensitivity were found with the target moving from one end to the other end of the sensor.
3. For the same target loading position, *S*_m_ decreases and tends to approach each other with the increase of viscous damping coefficient but the tendency becomes weak at high-order resonance.
4. The effect of viscous damping force on the decrease of *S*_m_ gradually reduces when the target approaches the node of the sensor.

## Figures and Tables

**Table 1:** Information for the sensor, liquid, and loading conditions in this study.

	Symbol	Unit	Value
Young’s modulus	*E*	GPa	105 [26]
Density	*ρ*	kg/m^3^	7.9 × 10^3^ [26]
Poisson’s ratio	*ν*		0.33 [26]
Length	*l*	mm	1
Width	*w*	mm	0.2
Thickness	*t*	μm	15
Loading position Viscous damping coefficient	*x_c_*/*l* *c*	− N/(m/s)	0, 0.05, 0.1, …, 1.0 0 (air) 80 (Liquid #1) 160 (Liquid #2) 240 (Liquid #3) 320 (Liquid #4)
